# Impact of Endometrial Preparation Protocols for Frozen Embryo Transfer on Live Birth Rates

**DOI:** 10.5041/RMMJ.10297

**Published:** 2017-04-28

**Authors:** Maria Cerrillo, Leyre Herrero, Alfredo Guillén, Mercedes Mayoral, Juan Antonio García-Velasco

**Affiliations:** IVI-Madrid, Rey Juan Carlos University, Madrid, Spain

**Keywords:** Frozen cycle, hormonal replacement therapy, IVF, natural cycle

## Abstract

**Background:**

It has been reported that a natural cycle (NC) is similar to or even better than hormone replacement therapy (HRT) in patients with regular cycles who undergo frozen embryo transfer (FET). Hundreds of FETs are managed yearly in our clinic. Scheduling these cycles is critical in a busy unit like ours. This is why we have to prove if a NC really shows a better outcome than other endometrium preparation protocols.

**Methods:**

Hence we carried out a prospective study between June 2011 and June 2012, which included 530 patients (570 FET cycles) randomly allocated to two study groups: Group 1 (*n*=280 cycles), artificial cycle (HRT); or group 2 (*n*=290 cycles), natural cycle. Natural cycles were later divided into two groups: 169 patients scheduled with human chorionic gonadotropin (hCG) and 121 with endogenous luteinizing hormone (LH) surge. The inclusion criteria were: age <39 years, regular menstrual cycles (26–35 days), and previous IVF cycle with embryo cryopreservation. The exclusion criteria were polycystic ovarian syndrome and endometriosis stage III/IV.

**Results:**

No statistical differences were found in the baseline characteristics among groups, nor between implantation or ongoing pregnancy rates (30.8% HRT group; 32.7% hCG group; 34.5% LH surge group). However, a higher miscarriage rate was observed in the HRT group when compared to hCG or LH surge (21.2% versus 12.9% versus 11.1%, *P*<0.01). Live birth rates were similar among groups, as were perinatal outcomes, for rates of natural delivery and weight and length of newborns.

**Conclusions:**

We conclude that scheduling FET with HRT on weekdays and avoiding work overload at weekends prove efficient and safe in cycle outcome terms. Another reason for the convenience of an HRT protocol is having fewer visits to the clinic compared to natural cycle protocols.

## INTRODUCTION

The first successful pregnancy from a frozen-thawed embryo transfer (FET) was reported in 1983.^1^ More than three decades have passed, and significant changes have been introduced into our clinical practice. Developments and improvements in cryopreservation methods have helped improve the efficiency of *in vitro* fertilization (IVF).^2^–^4^ These quantifiable modifications have led to vitrification being introduced as the main cryopreservation technique in almost every laboratory in the world. Vitrification was first described by Kuwayama et al.^5^ and has led to a cascade of major changes in the IVF practice worldwide.

The transfer of FET constitutes approximately 20% of all embryo transfers in IVF clinics; this percentage is increasing as the number of transferred embryos is lowered, and newer strategies to avoid ovarian hyperstimulation syndrome (OHSS) rely on a stable and efficient cryopreservation program.^3^

However, there is still some controversy as to the ideal endometrial preparation protocol. Different endometrial preparation strategies have been described. They include a purely natural cycle (NC) with LH detection in blood or urine; a natural modified cycle (NMC) in which hCG is administered to schedule embryo transfer instead of measuring LH; hormone replacement therapy (HRT) or artificial cycle with estradiol (E2) and progesterone (P4), with or without using gonadotropin-releasing hormone (GnRH) analogs; and, finally, stimulated cycles with low doses of gonadotropins.

The most recent meta-analysis does not support using one strategy over others,^6^ but other authors have supported the use of a NC with progesterone over the NC^7^ or the pure natural cycle over the NMC to report better results.^8^ The wide range of approaches in natural or artificial preparation, even within the same protocol, has also been demonstrated in surveys, which include 179 centers worldwide. Many different approaches can be found in answers about FET preparation and in questions such as if progesterone is needed, and if its timing in a natural or an artificial cycle shows a variety of responses.

After reviewing the literature and attempting to understand if one protocol was superior to the other two, we decided to run a prospective trial to compare the three groups based on our regular protocols (natural and modified natural with progesterone supplementation and an artificial cycle with no GnRH analog).

## MATERIALS AND METHODS

This prospective and observational cohort study was performed in IVI Madrid, a university-affiliated private clinic, between June 2011 and July 2012. This study was approved by our Institutional Review Board (MAD-MC-05-2011-01), and all the patients signed an informed consent before the procedure.

Women below the age of 40 with regular cycles (26–35 days) and no more than two previous IVF/intracytoplasmic sperm injection (ICSI) cycles were eligible for the study.

The exclusion criteria included irregular cycles, polycystic ovary syndrome (PCO) according to Rotterdam criteria,^9^ endometriosis stage III/IV, and preimplantation genetic diagnosis cycles. Egg donation FET was also excluded.

A total of 570 cycles from 530 patients were included and assigned to one of the three different interventions. Treatment allocation was carried out at the beginning of the cycle using a randomization list that was distributed among the clinicians involved in the study.

The artificial cycle treatment group included 280 cycles. In the NC group, 145 cycles were enrolled and monitored until a positive urine LH test was detected (121 cycles). In this group, the other 24 cycles did not show a positive urine LH test and were assigned to the NMC with hCG. In the NMC group, 145 cycles were initially enrolled, and the previous 24 cycles (negative LH test) were also added (169 cycles).

The cancellation rates (no embryo survival) were 9.6% (27 cycles), 4.1% (5 cycles), and 2.3% (4 cycles) in the artificial cycle, the NMC, and the NC, respectively.

### Endometrial Preparation Protocols

#### Hormone Replacement Therapy (E2+P4)

Endometrial preparation was initiated with oral estradiol valerate (Progynova®; Schering, Madrid, Spain) on cycle day 2–3 after a transvaginal ultrasound. The classic endometrium build-up preparation protocol started with 2 mg/day on days 1–4, 4 mg/day on days 5–8, and 6 mg/day from day 9 onward. A second transvaginal ultrasound was performed after 10–12 days of estrogen treatment. If endometrial thickness was at least 6 mm, and there was no dominant follicle, nor signs of ovulation, embryo transfer was scheduled. Natural micronized progesterone was vaginally administered (Utrogestan®; Seid, Madrid, Spain) at a dose of 400 mg/12 h for 3 or 5 complete days before embryo transfer, depending on the cleavage stage of embryos (embryo age +0 days). Progesterone supplementation continued if pregnancy occurred until 12 weeks of pregnancy.

#### Natural Cycle Triggered by hCG

A series of ultrasound examinations was performed after spontaneous menses in this group of patients to detect the dominant follicle. Once a mean diameter of 17 mm had been reached and minimal endometrial thickness was 6 mm, hCG (Ovitrelle®, 250 μg; Merck, Madrid, Spain) was administered subcutaneously that evening, and embryos were thawed and transferred 5 or 7 days later according to embryo stage (5 days for day-3 embryos, and 7 days for day-5 blastocysts). Luteal phase support commenced 3 or 5 days before embryo transfer (embryo age +0 days) by administering micronized vaginal progesterone at a dose of 200 mg/12 h daily (Utrogestan®, 200 mg; Seid, Barcelona, Spain) until week 5 of pregnancy.

#### Natural Cycle with Spontaneous LH Surge

Vaginal ultrasound monitoring was performed after spontaneous menstruation. Based on the ultrasound examinations, the patient was instructed to start monitoring morning urinary LH (Ovulation test, Clearblue®; Swiss Precision Diagnostics, Geneva, Switzerland) when the mean diameter of the leading follicle was at least 14 mm. Although urinary LH is not accurate enough to determine the exact timing of ovulation, it is good enough to provide a good estimate and extremely convenient for the patients, as for practical issues we could not bring them to the clinic for serum LH on a daily basis. The minimal endometrial thickness had to be 6 mm to perform FET. Embryo transfer was scheduled 5–7 days after detecting the LH surge according to the frozen embryo age. Day-3 embryos were transferred 5 days after the LH surge, and day-5 embryos were transferred 7 days after LH surge. Luteal phase support was started by administering micronized vaginal progesterone at a dose of 200 mg/12 h daily (Utrogestan®, 200 mg; Seid, Barcelona, Spain) 3 or 5 days before embryo transfer (embryo age +0 days) until week 5 of pregnancy.

### Embryo Cryopreservation

The Cryotop® method was used to vitrify and rewarm surplus embryos, as described by Kuwayama et al.^5^ and Cobo et al.^10^ All the required materials were obtained from Kitazato (Tokyo, Japan). After warming the embryos to be transferred, confirmation was obtained that at least 50% of blastomeres were intact. With blastocysts, a re-expansion evaluation was made 2 h after thawing and after confirming that an inner cell mass was present.

### Embryo Transfer

Following our institution protocol of the standardization of procedures, embryo transfer was performed using a Cook catheter (K-SOFT-5000 Soft-Trans; Cook Ob/Gyn, Cork, Ireland) with ultrasound guidance.

### Outcomes

The main outcome was the ongoing pregnancy rate, defined as at least one viable fetus beyond gestation week 12 by ultrasound. Secondary outcomes were implantation rate, biochemical pregnancy rate (defined as a positive pregnancy test), clinical pregnancy rate (defined as the presence of a gestational sac with an embryo with a positive heartbeat), and miscarriage rate (pregnancy loss until week 12 of gestation after previously confirming a clinical pregnancy by ultrasound).

### Sample Size Calculation

Based on the results of earlier studies^11^ and our center’s pregnancy rates, we calculated that we would need 220 patients to detect at least a 10% difference in the pregnancy rate with an 80% power at a 5% two-tailed statistical level of significance.

### Statistical Analysis

The statistical analysis was performed using version 17 of the Statistical Package for Social Sciences (SPSS, Chicago, IL, USA). A chi-square and *t* test, or an analysis of variance (followed by the Bonferroni *post hoc* test), were used to compare proportions or means among groups. *P* values of <0.05 were considered significant.

## RESULTS

The results obtained in the three study groups are shown in Table 1. Between June 2011 and July 2012, 570 cycles were included in the study. In the HRT group, 253 cycles were finally analyzed, with 165 in the NMC and 116 in the NC. The ages of the patients enrolled in this study were comparable.

**Table 1:** Results of the Thawing Cycles.

	HRT	hCG	LH Surge	*P* Value
Cycles, *n*	280	169	121	
Embryo transfers, *n*	253	165	116	
Age, years, mean±SD	35.5±2.7	35.3±2.3	35.4±2.6	NS
Embryos transferred, mean±SD	1.6±0.5	1.5±0.7	1.5±0.6	NS
Pregnancy rate, % (*n*)	47.4 (120)	45.4 (75)	46.5 (54)	NS
Clinical pregnancy rate, % (*n*)	39.1 (99)	37.5 (62)	38.8 (45)	NS
Implantation rate, %	26.9	31.4	31.2	NS
Ongoing pregnancy rate, % (*n*)	30.8 (78)	32.7 (54)	34.5 (40)	NS
Live birth rate, % (*n*)	36.7 (93)	41.2 (68)	43.1 (50)	NS
Miscarriage rate, % (*n*)	21.2 (21)^a^	12.9 (8)^b^	11.1 (5)^c^	a versus b: 0.01 a versus c: 0.01

Note: Data are expressed as mean±SD, and as percentage (*n*).

hCG, human chorionic gonadotropin; HRT, hormone replacement therapy; LH, luteinizing hormone; NS, not significant.

No statistical differences were found among the groups in terms of pregnancy, implantation, and ongoing pregnancy rates. A higher miscarriage rate in the HRT group of 21.2% was observed compared to the other two groups (12.9% for the hCG group and 11.1% for the LH surge group).

The obstetrics and perinatal results of these ongoing pregnancies are shown in Table 2. In the HRT group, 84.6% (66/78) were singleton versus 15.3% (12/78) twin pregnancies; in the hCG group 83.3% versus 16.6%, and NC 75% versus 25%. The percentages of natural delivery and cesarean sections were similar in the three study groups. The mean gestational age at delivery in the singleton pregnancies was also comparable in the three groups. The weight and length of single newborns were similar in the three groups, but higher values than expected were obtained for the mean weight and length of newborn twins.

**Table 2:** Obstetrics Outcomes.

	HRT	hCG	LH Surge	*P* Value
Ongoing pregnancies, *n*	78	54	40	
Live birth rate, *n*	93	68	50	
Singleton ongoing pregnancies, % (*n*)	84.6 (66)	83.3 (45)	75.0 (30)	NS
Vaginal delivery, % (*n*)	66.6 (42/63)	66.7 (30/45)	53.3 (16/30)	NS
Cesarean section, % (*n*)	33.3 (21/63)	33.3 (15/45)	46.6 (14/30)	NS
Gestational age, mean±SD	38.3±2.8	38.4±3.1	39.0±3.5	NS
Fetal weight, mean±SD	3254.6±652.2	3236.1±647.3	3219.4±524.6	NS
Fetal length, mean±SD	51.2±2.6	49.8±3.1	51.5±2.1	NS
Twin ongoing pregnancies, % (*n*)	15.3 (12/78)	16.6 (9/54)	25 (10/40)	NS
Vaginal delivery, % (*n*)	13.3 (2/12)	33.3 (3/9)	10.0 (1/10)	NS
Cesarean section, % (*n*)	83.3 (10/12)	66.6 (6/9)	90.0 (9/10)	NS
Gestational age, mean±SD	34.3±2.6	34.6±2.9	35.6±2.6	NS
Fetal weight, g, mean±SD	1966.2±292.3	2050.9±325.5	2096.3±358.1	NS
Fetal length, cm, mean±SD	46.3±2.1	49.2±2.6	45.8±2.5	NS

hCG, human chorionic gonadotropin; HRT, hormone replacement therapy; LH, luteinizing hormone; NS, not significant.

## DISCUSSION

This study suggests that preparing FET with an artificial cycle, an hCG-triggered NC with luteal progesterone, or a pure NC after spontaneous ovulation with luteal support results in similar implantation rates, clinical and ongoing pregnancy rates, and live birth rates.

Our findings are similar to results from previous Cochrane reviews.^12^ The first study with seven randomized controlled trials, including six different interventions, was studied, and we focused on analyzing only the comparison between estrogen and progesterone FET versus NC FET. This comparison was reduced to one randomized controlled trial (RCT) published in 1994 that included 100 patients.^13^ The clinical pregnancy rate per woman did not differ between the two treatments (OR 1.06, 95% CI 0.40–2.80, *P*=0.91). In the Cochrane review and a meta-analysis,^12^ which included prospective (those of Ghobara^12^) and retrospective trials, it was concluded that no single method for FET proved more effective than others. A randomized controlled trial, cited in the review by Groenewoud, was performed by Weissman et al.,^14^ who enrolled 60 ovulatory women. They were randomized to FET after spontaneous ovulation or a hCG-triggered cycle. Luteal phase support was used in both groups. They reported no significant differences in the implantation, pregnancy, or live birth rates.

In a retrospective study of more than 4,000 cycles, which was also included in the review by Groenewoud et al.,^6^ the NC was compared with luteal progesterone supplementation, the NC with hCG for ovulation induction without progesterone (which differentiates it from our groups), and also the substituted cycle (estradiol and progesterone). The authors found a higher positive pregnancy test rate in the last-mentioned group, but reported comparable clinical pregnancy and delivery rates in the three protocols.

The use of progesterone supplementation in the NC or NMC is still a matter of debate. In an RCT with 435 patients,^7^ the live birth rate was significantly higher in the group with progesterone compared to FET after an NC than it was in the group that did not receive it (30% versus 20%, *P*=0.0272).

Nevertheless, another paper on the use of progesterone in the luteal phase of FET after hCG triggering (an NMC) was unable to provide compelling evidence.^16^

In our study the clinical pregnancy rates were similar in all three groups (39.1%, 37.5%, and 38.8%). However, a higher miscarriage rate was observed in the artificial preparation group (21%) compared with NCs (12%) or NMCs (11%). Although these pregnancy losses had no effect on the ongoing pregnancy rate as regards a significant difference (30%, 32%, and 35%), this finding deserves further consideration.

This higher miscarriage rate observed in the patients with an artificial cycle agrees with the results of two previous studies, which both reported higher rates of biochemical pregnancies and higher miscarriage rates in the artificial groups. The retrospective observational study by Guillen et al.^17^ was performed in all the IVI group clinics from February 2010 to March 2011, and 3,027 artificial cycles were compared to 1,498 natural cycles. The positive pregnancy test rate (48.8% versus 45.3%), the clinical pregnancy rate (40.9% versus 37.7%), and the miscarriage rate (9% versus 5.8%) were significantly higher for FET with artificial preparation than for NC. In the group of artificially prepared cycles, patients with irregular cycles were included. This inclusion criterion differed from our population with regular cycles. The other retrospective study^16^ involved 4,470 FET cycles. In HRT cycles a significantly higher pregnancy loss (41.5%) was observed compared to natural ones with progesterone (22.4%) or to NMCs (33.6%). In this retrospective series, the artificial group included more patients with PCO and described higher miscarriage rates, which were also found in our study despite our patients being young with regular cycles and prospectively selected.

In 2010 Fatemi et al. reported^8^ that NC FET with spontaneous ovulation was superior to an NMC with hCG. This study, a prospective randomized trial with 61 and 63 patients, showed higher ongoing pregnancy rates in NC FET with spontaneous ovulation than in cycles programmed with hCG. Nevertheless, a comparison with our study is limited by the fact that progesterone supplementation was not used in that trial. This group was based on a possible negative effect of hCG on the endometrium, but this series included fewer patients. No other studies have results similar to that of Fatemi (i.e. better results for NC FET versus NMC).^18^,^19^ For example, Weissman et al. in his retrospective series published in 2009,^20^ and the prospective study in 2011,^14^ published similar rates for both groups.

However, it is still unclear if hCG has a negative impact on endometrial receptivity,^15^ and we hope that new technologies, including endometrial receptivity arrays, will help identify any existing differences between protocol types, or will be able to determine whether or not hCG impacts endometrial receptivity.

While the higher miscarriage rates in artificial cycles have been elucidated, data on live birth rates are reassuring with different protocols.

In busy IVF units the efforts of clinic staff should also focus on programming cycles and distributing workloads across the seven weekdays. Chaotic transfer activity and cryopreservation unit overloads during certain periods of time could lead to reduced quality standards and, consequently, to a drop in the laboratory’s pregnancy rates.

Other small units with no activity at weekends could also find artificial cycles appealing, which would allow FETs to be programmed on weekdays.

Natural cycles and NMCs could be more convenient to patients as no hormonal therapy is applied for several weeks.

The disadvantages of monitoring NCs or NMCs have been studied by Weissman et al. who compared the number of clinical visits and cycle outcomes.^15^ They showed that triggering ovulation with hCG is as efficient as serial monitoring until ovulation is detected in patients. As ovulation triggering by hCG significantly reduces the number of monitoring visits needed to schedule the day of FET, this approach may be superior in terms of patient convenience and the cycle’s cost-effectiveness.^14^

Patients who undergo an artificial cycle are easily monitored by ultrasound at the beginning of the cycle, and another is done 10–14 days later. Cancellations due to spontaneous ovulations are also infrequent despite the fact that no GnRH analog is used. It is, therefore, an easy protocol that does not involve many monitoring visits and one that makes planning for both local and long-distance patients feasible.

Endometrium preparation can be completed with a variety of protocols. Freeze-all strategies to prevent ovarian hyperstimulation syndrome with agonist triggering, high progesterone level on the day of hCG, and blastocyst biopsy in chromosome-comprehensive screening cycles have also increased the number of FETs in clinics.

Preparing the endometrium for FET would be identical to preparing for an egg donation cycle with fresh or vitrified oocytes and preparing a cycle with own vitrified oocytes. Similar perinatal data are obtained (percentage of normal delivery or cesarean sections, gestational age at birth, weight and length of neonates) with the three endometrial preparation protocols in accordance with a recently published paper.^21^ There is an urgent need to elucidate the best method to prepare the endometrium for all these indications in terms of live birth rates, and one that is also convenient for patients and IVF clinics.

## CONCLUSION

We conclude that in patients with regular cycles who undergo FET, an NC with urinary LH monitoring, or with hCG, may be a good option that has no adverse effects on cycle outcome. Absence of further hormonal therapy in NC protocols may be another argument that would make it appealing to women with regular cycles. An artificial cycle with the estrogen and progesterone combination is a more flexible and convenient protocol for patients with irregular cycles, but has achieved similar results to other endometrium preparation protocols used in women with regular cycles. The HRT protocol may be more convenient given the fewer monitoring visits involved than natural protocols, and also given the possibility of scheduling FETs on weekdays.

## Abbreviations

E2: estradiol
FET: frozen-thawed embryo transfer
GnRH: gonadotropin-releasing hormone
hCG: human chorionic gonadotropin
HRT: hormone replacement therapy
IVF: *in vitro* fertilization
LH: luteinizing hormone
NC: natural cycle
NMC: natural modified cycle
P4: progesterone
PCO: polycystic ovary syndrome
RCT: randomized controlled trial
